# Kidney function of HIV-infected children in Lagos, Nigeria: using Filler's serum cystatin C-based formula

**DOI:** 10.1186/1758-2652-13-17

**Published:** 2010-05-18

**Authors:** Christopher I Esezobor, Edna Iroha, Olajumoke Oladipo, Elizabeth Onifade, Oyetunji O Soriyan, Adebola O Akinsulie, Edamisan O Temiye, Chinyere Ezeaka

**Affiliations:** 1 Department of Paediatrics, College of Medicine, University of Lagos and Lagos University Teaching Hospital, Idi-Araba, Lagos State, Nigeria; 2 Laboratory and Genomic Medicine, Department of Pathology and Immunology, Washington University School of Medicine, St Louis, MO, USA; 3 Children's Unit, Friarage Hospital, Northallerton, North Yorkshire, DL6 1JG, UK; 4 Department of Clinical Pathology, College of Medicine, University of Lagos and Lagos University Teaching Hospital, Idi-Araba, Lagos State, Nigeria

## Abstract

**Background:**

Limited data is available on kidney function in HIV-infected children in sub-Saharan Africa. In addition, malnutrition in these children further reduces the utility of diagnostic methods such as creatinine-based estimates of glomerular filtration rate. We determined the serum cystatin C level and estimated glomerular filtration rate of 60 antiretroviral-naïve, HIV-infected children and 60 apparently healthy age and sex matched children.

**Methods:**

Serum cystatin C level was measured using enzyme-linked immunosorbent assay technique, while glomerular filtration rate was estimated using Filler's serum cystatin C formula. Student t test, Mann Whitney U test, Pearson chi square and Fisher's exact test were used, where appropriate, to test difference between groups.

**Results:**

Compared to the controls, the HIV-infected group had significantly higher median (interquartile range) serum cystatin C levels {0.77 (0.29) mg/l versus 0.66 (0.20) mg/l; p = 0.025} and a higher proportion of children with serum cystatin C level >1 mg/l {10 (16.7%) versus one (1.7%); p = 0.004}. The HIV-infected children had a mean (± SD) eGFR of 96.8 (± 36.1) ml/min/1.73 m^2 ^compared with 110.5 (± 27.8) ml/min/1.73 m^2 ^in the controls (p = 0.021). After controlling for age, sex and body mass index, only the study group (HIV infected versus control) remained a significant predictor of serum cystatin C level (β = -0.216, p = 0.021). The proportion of HIV-infected children with eGFR <60 ml/min/1.73 m^2 ^was eight (13.3%) versus none (0%) in the control group (p = 0.006). However, the serum cystatin C level, eGFR and proportions of children with serum cystatin C level >1 mg/l and eGFR <60 ml/min/1.73 m^2 ^were not significantly different between the HIV-infected children with advanced disease and those with milder disease.

**Conclusions:**

HIV-infected children in Nigeria have higher serum cystatin C level and lower eGFR compared to age and sex matched controls.

## Background

Although sub-Saharan Africa has the largest number of children living with HIV, little is known about the prevalence of HIV-related kidney disease in these children despite the recognition of HIV infection as a strong initiation risk factor for kidney disease [1,2]. The dearth of resources for care of children with kidney diseases and the limited choice of antiretroviral drugs in Africa underline a need to not only accurately assess glomerular filtration rate (GFR) in these children, but to do so early if their kidney function is to be preserved.

However, the well-known method of assessment of kidney function using creatinine-based formulae is fraught with several shortcomings in the general population [3,4] and particularly in HIV-infected persons [5]. Notably, creatinine clearance is significantly influenced by tubular secretion [3,4] and the serum creatinine level is affected by non-renal factors, such as diet, race and lean mass. Also, the only method widely available for clinical analysis of creatinine in Nigeria is the modified kinetic Jaffe method with its accompanying limitations [6].

Moreover, malnutrition is common among children in sub-Saharan Africa, especially in those living with HIV/AIDS [7]. This further reduces the usefulness of serum creatinine-based formulae in the routine assessment of kidney function and has implications for early detection of impaired kidney function in these children [8].

Conversely, cystatin C, a 13 kilo dalton 120-amino acid peptide produced at a fairly constant rate by the house-keeping genes of all nucleated cells, has been shown to be a better measure of glomerular filtration [8-10]. Compared to creatinine, it is less affected by non-glomerular factors, such as lean mass, diet and tubular secretion [3,9,10]. Similarly, cystatin C-based formulae for estimation of GFR closely mirror gold-standard measures of GFR and reflect changes in GFR earlier than creatinine-based formulae [8,11]. Of the several cystatin C-based formulae for GFR, the one proposed by Filler *et al *[11], developed from a study involving children between the ages of one year and 18 years, has been shown to be of greater precision, lower bias and higher accuracy than the creatinine-based formula of Schwartz and most other cystatin C-based formulae [12].

In the present study, we compared kidney function in children with and without HIV infection attending the outpatient clinics of the Lagos University Teaching Hospital using the serum level of cystatin C, and estimated GFR using Filler's cystatin C-based formula.

## Methods

### Study setting and population

The study was done between January and June 2008, and was approved by the Hospital's Research and Ethics Committee. It was a cross-sectional observational study of HIV-infected children attending the Paediatric Special Clinic of the Lagos University Teaching Hospital. The clinic, which provides comprehensive antiretroviral therapy, is largely supported by the US President's Emergency Plan For AIDS Relief (PEPFAR).

The diagnosis of HIV infection was based on a documentary evidence of HIV infection using the enzyme-linked immunosorbent assay (ELISA) test and confirmed by western blot technique; for those who were diagnosed before the age of 18 months, diagnosis was based on positive HIV DNA PCR tests on two separate samples. All consecutive HIV-infected antiretroviral-naïve children, aged 18 months to 16 years and who met the study criteria, were eligible. Excluded from the study were children: with sickle cell disease, with cardiac disease or previously confirmed kidney disease; those hospitalized within the past two weeks; those with the presence of any illness severe enough to require hospitalization; those with diarrhoea; and those who had used systemic steroids within the past one week.

During the study time frame, 71 antiretroviral-naïve HIV-infected children attended the Paediatric Special Clinic, and 11 were excluded: six needed immediate hospitalization for acute illnesses; three samples were discarded because of haemolysis; and two had incomplete CD4+ cell count or percentage results. The excluded children were not different in age and sex distribution from those enrolled. A one-to-one age and sex matched pairing was done from a pool of HIV-uninfected children. Age matching was done to the nearest half year for those ≤ 5 years and to the nearest year for those older than five years. Where more than one control existed, a ballot was taken. The matching was done prior to analysis of the sample for serum cystatin C to avoid bias.

The controls were ambulatory, apparently healthy children recruited while attending other clinics of the hospital: respiratory clinic (n = 17, those with bronchial asthma, but not receiving systemic steroid or those being followed up after hospitalization for bronchopneumonia); neurology clinic (n = 27, children with one or more episodes of seizure referred to the clinic, but not yet on anticonvulsants); surgical clinic (n = 11, children prior to or after herniotomy, post appendicectomy); and ear, nose and throat clinic (n = 5, those with speech or hearing disorders).

No incentive to participate in the study was offered to the caregivers. The inclusion and exclusion criteria were the same for both groups of participants, except for the absence of HIV infection in the control group.

### Data collection

After informed consent, relevant clinical and demographic data were obtained by interviewing each caregiver and physically examining each child. The clinical notes and chest X-rays of each participating child with HIV infection were also reviewed for a diagnosis of tuberculosis. The data obtained were used in staging the HIV disease, according to the revised World Health Organization (WHO) paediatric clinical staging criteria [13].

About 5 ml of blood was obtained from each participating child. Out of this, 2 ml was allowed to clot, and the resulting serum after centrifugation was frozen at -80°C until analysis. The remaining 3 ml of blood was used for haemoglobin electrophoresis and CD4+ cell count or percentage in the HIV-infected children and in the controls for haemoglobin electrophoresis and detection of HIV antibodies (using Determine HIV rapid kit™ manufactured by Abbot Japan Co Ltd for Inverness Medical Japan Co Ltd).

In batches of 30 samples, the serum cystatin C was measured by a sandwich enzyme-linked immunosorbent assay for the quantitative measurement of human cystatin C, using kits manufactured by BioVendor - Laboratorni medicina a.s, Czech Republic. The intra-assay co-efficient of variation (CV) for the quality controls (QC) were as follows: low QC, 3.9%; high QC, 3.1%. The inter-assay CV were as follows: low QC, 6.8% and high QC, 11.8%.

The estimated glomerular filtration rate (eGFR) (ml/min/1.73 m^2^) was derived using a cystatin C-based formula as proposed by Filler *et al *[11], i.e.

where 1/cystatin C is the reciprocal of the concentration of serum cystatin C in mg/l.

Advanced HIV disease was defined clinically as WHO Clinical Stage 3 disease or Clinical Stage 4 disease, or immunologically as CD4+ cell count <350 cells/mm^3 ^(in those ≥ 5 years old) or CD4 percentage 20% (in those younger than five years). Of the 35 children with advanced HIV disease, 10 (28.6%) were classified based on WHO clinical criteria only, 10 (28.6%) on immunological criteria only, and 15 (42.9%) on both clinical and immunological criteria.

### Statistical analysis

The data were analyzed using SPSS version 14.0 (SPSS for Windows Inc., Chicago, IL, USA) statistical software. Anthropometric z scores were calculated using the WHO AnthroPlus software developed using the WHO Child Growth Standards and the WHO Reference 2007 [14]. Continuous data were summarized as mean (± SD) and median (inter-quartile range) for parametric and non-parametric data, respectively, while categorical data were represented as proportions. Pearson's Chi square or Fisher's exact test was used to compare categorical data, while Mann Whitney U test and Student's t test were used to analyse the non-parametric (serum cystatin C) and parametric (eGFR) data, respectively.

A multiple linear regression, simultaneously accounting for weight, height, age, gender and study group (HIV positive versus control) with log transformed cystatin C as the dependent variable, was performed (Model 1). A separate model (Model 2) was tested, using age, sex, study group and body mass index (BMI) as independent variables to avoid colinearity between BMI and weight and height. Differences, correlations (Spearman's) and regression coefficient between variables were considered significant if the p value was less than 0.05.

## Results

Table 1 displays the results of the HIV-infected children compared with the controls. Sixty children with HIV infection and 60 apparently healthy, HIV-uninfected children were studied. The median age was 5.5 (5.2) years in the HIV-infected group and 5.3 (5.2) years in the control. The HIV-infected children were leaner (BMI z score -1.07 vs. -0.31; p = 0.048), shorter (HAZ score -1.00 vs. 0.05; p = 0.000) and weighed less (WAZ score -1.30 vs. -0.15; p = 0.000) compared with children in the control group.

**Table 1:** Demographic and kidney-related characteristics of study participants.

Characteristics	HIV-infected children (n = 60)	Control (n = 60)	p value
Age (years)			
Median (IQR)	5.5 (5.2)	5.3 (5.2)	0.990^a^
<5 years, n (%)	28 (46.7)	26 (43.3)	
Male gender, n%	35 (58.3)	35 (58.3)	1.00^b^
Weight			
Median (kg)	15.3 (8)	19.8 (11.9)	0.026^a^
WAZ score	-1.30	-0.15	0.000^a^
Length/height			
Median (cm)	107 (25)	115 (36)	0.181^a^
HAZ score	-1.00	0.05	0.000^a^
BMIZ score	-1.07	-0.31	0.048^a^
Blood pressure, mean (mmHG)	66.9 (10.9)	64.9 (7.7)	0.223^c^
Serum cystatin C			
median (mg/l)	0.77 (0.29)	0.66 (0.20)	0.025^a^
>1.0 mg/l, no. (%)	10 (16.7)	1 (1.7)	0.004^b^
Estimated GFR			
mean (ml/min/1.73 m^2^)	96.8 (36.1)	110.5 (27.8)	0.021^c^
<60 ml/min/1.73 m^2^, no. (%)	8 (13.3)	0 (0)	0.006^b^

WAZ: weight for age z score; HAZ: height for age z score; BMIZ: Body mass index z score; ^a^Mann-Whitney U test; ^b^Pearson's Chi square test; ^c ^Student t test; IQR: interquartile range

The median serum cystatin C level was 0.77 (0.29) mg/l in the HIV-infected group compared with 0.66 (0.20) mg/l in the control (p value = 0.025).

There was no statistically significant difference in the cystatin C level of the controls recruited from the various clinics (result not shown). Similarly, 10 (16.7%) children with HIV infection compared with one in the control group (1.7%) had a serum cystatin C level >1.0 mg/l (p value = 0.004). The mean eGFR was 96.8 (± 36.1) ml/min/1.73 m^2 ^in the children with HIV infection and was significantly lower than the eGFR of 110.5 (± 27.8) ml/min/1.73 m^2 ^in the control group (p = 0.021). Conversely, eight (13.3%) of the children with HIV infection, compared with none in the control group, had an eGFR less than 60 ml/min/1.73 m^2 ^(p = 0.006).

Table 2 displays the characteristics of the children with HIV infection. In children younger than five years of age, the median CD4 percentage was 12.6% in those with advanced disease versus 25.1% in those with less advanced disease. In children five years or older, the median CD4 count was 343 cells/mm^3 ^in those with advanced disease versus 568 cells/mm^3 ^in those with less advanced disease (p = 0.002). There was no significant difference in cystatin C level, eGFR and proportions with serum cystatin C >1 mg/l and eGFR <60 ml/min/1.73 m^2 ^between children with advanced disease versus the less advanced disease, although most measures of kidney function were worse in the advanced disease group.

**Table 2:** Serum cystatin C and eGFR of children with different stages of HIV disease.

Variables	Stage of HIV disease		p value
	Advanced n = 35	Not advanced n = 25	
HIV-related variables:			
For <5 years, CD4%, median (IQR)	12.6 (8.2)	25.1 (5.2)	0.000^a^
For ≥ 5 years, CD4 count/mm^3^, median(IQR)	343 (488)	568 (507)	0.002^a^
Serum cystatin C, median (mg/l)	0.79 (0.35)	0.76 (0.24)	0.196^a^
Cystatin C >1.0 mg/l, no. (%)	8 (22.9)	2 (8)	0.119^b^
eGFR, mean (ml/min/1.73 m^2^)	97.3 (4.2)	96.2 (23.6)	0.904^c^
eGFR <60 ml/min/1.73 m^2 ^no. (%)	6 (17.1)	2 (8)	0.265^b^

^a ^Mann Whitney U test; ^b ^Pearson's Chi square test; ^c ^Student's t test

**Figure 1:**
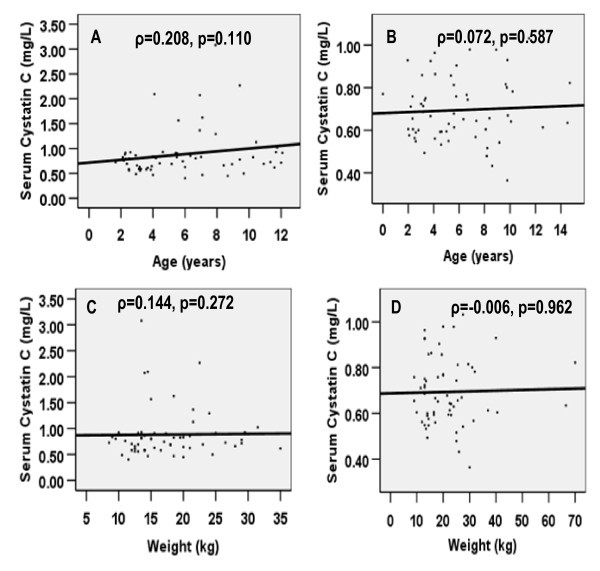
**Correlation of serum cystatin C with age and weight in HIV-infected children (A & C) and in the controls (B & D)**.

**Figure 2:**
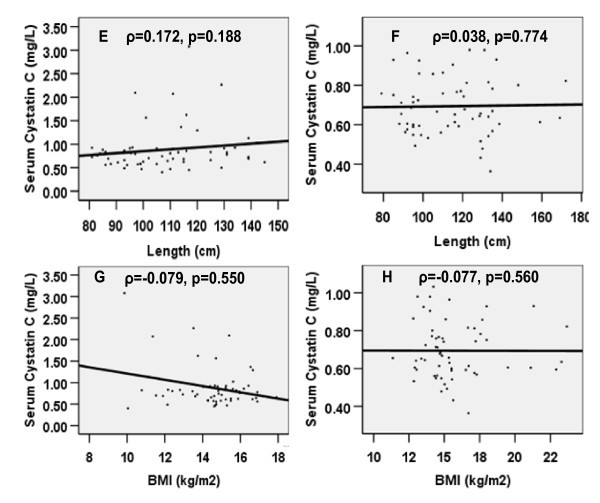
**Correlation of serum cystatin C with length and BMI in HIV-infected children (E & G) and in the controls (F & H)**.

The correlation of weight, length, BMI and age with serum cystatin C was small and not significant in both groups of children (Figure 1 &2). In multiple linear regression, the variation in cystatin C as explained by both models was small and only reached significant proportion in Model 2 (model including BMI), Table 3 and Table 4. Only the study group (HIV infected versus control) remained a significant predictor of cystatin C after controlling for other variables, including BMI.

**Table 3:** Multiple linear regression model simultaneously accounting for age, gender, length, weight and study group (Model 1).

Independent variables	Unstandardized coefficient	Standardized coefficient	p value
Constant	-0.067	-	0.686
Age	0.016	0.341	0.173
Gender	-0.009	-0.03	0.741
Length	-0.001	-0.07	0.817
Weight	-0.003	-0.205	0.301
Study group	-0.055	-0.186	0.065

R^2 ^= 0.089, p = 0.057

**Table 4:** Multiple linear regression model simultaneously accounting for age, gender, study group and BMI (Model 2).

Independent variables	Unstandardized coefficient	Standardized coefficient	p value
Constant	-0.019	-	0.844
Age	0.006	0.129	0.155
Gender	-0.010	-0.034	0.711
BMI	-0.007	0.111	0.252
Study group	-0.063	-0.216	0.0210

R^2 ^= 0.083 p.value = 0.039

## Discussion

The study documented a high level of serum cystatin C and significant reduction in eGFR in HIV-infected children stable enough to attend the outpatient clinic of the Lagos University Teaching Hospital. Though the mean eGFR was within the normal range, more HIV-infected children than controls had eGFR less than 60 ml/min/1.73 m^2^. Similarly, more HIV-infected children than controls had a serum cystatin level greater than 1 mg/l, a level associated with an increased risk of death from cardiovascular and kidney diseases in elderly adults [15,16].

The elevated serum cystatin C level in HIV-infected children in this study is consistent with the reports of the Fat Redistribution and Metabolic Change in HIV infection (FRAM) study [5], the Nutrition for Healthy Living Study [8] and Jaroszewicz *et al *[17]. The high serum cystatin C level among the children with HIV infection may imply a significant reduction in glomerular filtration because, unlike creatinine, glomerular filtration is the only significant means of the plasma clearance of cystatin C [10,18]. HIV infection, by inducing a glomerulopathy (as exemplified in HIV-associated nephropathy), results in a reduction in the plasma clearance of substance by glomerular filtration [2].

In agreement with results from the majority of studies in this field [9,19-21], there was no significant correlation of serum cystatin C with age, sex, length, BMI and weight. Only the study group remained a significant predictor of cystatin C. This is consistent with the report of a recent study in children [21], which did not find any association between cystatin C and body mass, although studies involving adults have revealed varied results [19,22,23].

Our finding of high prevalence of eGFR less than 60 ml/min/1.73 m^2 ^in the HIV-infected children is consistent with a previous study [24], which documented a proteinuria prevalence of 20.5% among this clinic cohort of HIV-infected children. Similarly, a large body of research [25-27] have documented high prevalence of kidney diseases in HIV-infected persons. Jones *et al *[8] and Wools-Kaloustian and colleagues [27] reported prevalence of GFR less than 60 ml/min/1.73 m^2 ^between 11.5% and 15.2%, respectively; these findings are similar to ours.

The high level of serum cystatin C and prevalence of GFR less than 60 ml/min/1.73 m^2 ^in this cohort of HIV-infected children in Nigeria supports the association between HIV infection and kidney disease [2,25], and implies that HIV-related kidney disease may be as common in African children as in children residing in other regions of the world [24-26].

The significant reduction in GFR among HIV-infected children stable enough to attend outpatient clinics probably indicates a chronic, rather than a rapidly evolving, reduction in GFR. In sub-Saharan Africa, where the burden of HIV infection is high and the dearth of resources for kidney care profound, the high prevalence of glomerular dysfunction in HIV-infected children documented in this study warrants early detection of kidney involvement in HIV infection and institution of measures that may halt progression to end-stage kidney disease.

In donor-driven programmes (prevalent in the region) where the choice of antiretroviral drugs is limited, the high proportion of HIV-infected children with GFR less than 60 ml/min/1.73 m^2^, a GFR level that may require adjustment of drug dosages, is of concern. It also requires caution when rolling out antiretroviral drug regimens, including indinavir, adefovir and tenofovir that affect kidney function [5,28].

The lack of a significant difference between the GFR in HIV-infected children with advanced stage of HIV disease compared with those with less advanced stage could be due to the criteria chosen for classification of HIV disease into "advanced" and "not advanced" groups. Also, the small sample size could have underpowered the detection of any significant difference in eGFR and serum cystatin C.

The inclusion of only antiretroviral-naïve, HIV-infected children is a strength of this study because it provided an opportunity to document the prevalence of reduced GFR not confounded by the effects of highly active antiretroviral therapy (HAART). To our knowledge, this is the first study to do so. Published reports [5,8,17] of cystatin C or cystatin C-based eGFR in HIV-infected persons included those on HAART with regimens consisting of tenofovir and indinavir, and the reported increase in serum cystatin C in these studies may have been confounded by the use of these drugs [5,28]. We also analyze serum cystatin C in a single laboratory, which helps to reduce systematic error.

Our limitations include estimation of GFR, rather than actual measurement and unavailability of C-reactive protein, which has been shown to positively correlate with cystatin C level [29]. However, a previous report [24] of high prevalence of kidney disease in this clinic cohort of HIV-infected children strengthens the validity of our finding.

In conclusion, we have reported an elevated serum cystatin C level unaffected by BMI and a high prevalence of GFR <60 ml/min/1.73 m^2 ^in antiretroviral-naïve, HIV-infected children attending the outpatient clinic of the Lagos University Teaching Hospital.

## Competing interests

The authors declare that they have no competing interests.

## Authors' contributions

CIE and OO conceived the study and, with the other authors, participated in the design, data collection, analysis and interpretation of the results. All authors approved the final draft of the work. Personal funds were used for this study.
